# Dynamical analogues of rank distributions

**DOI:** 10.1371/journal.pone.0211226

**Published:** 2019-02-04

**Authors:** Carlos Velarde, Alberto Robledo

**Affiliations:** 1 Instituto de Investigaciones en Matemáticas Aplicadas y en Sistemas, Universidad Nacional Autónoma de México, Mexico City, Mexico; 2 Instituto de Física y Centro de Ciencias de la Complejidad, Universidad Nacional Autónoma de México, Mexico City, Mexico; Universidad Rey Juan Carlos, SPAIN

## Abstract

We present an equivalence between stochastic and deterministic variable approaches to represent ranked data and find the expressions obtained to be suggestive of statistical-mechanical meanings. We first reproduce size-rank distributions *N*(*k*) from real data sets by straightforward considerations based on the assumed knowledge of the background probability distribution *P*(*N*) that generates samples of random variable values similar to real data. The choice of different functional expressions for *P*(*N*): power law, exponential, Gaussian, etc., leads to different classes of distributions *N*(*k*) for which we find examples in nature. Then we show that all of these types of functions can be alternatively obtained from deterministic dynamical systems. These correspond to one-dimensional nonlinear iterated maps near a tangent bifurcation whose trajectories are proved to be precise analogues of the *N*(*k*). We provide explicit expressions for the maps and their trajectories and find they operate under conditions of vanishing or small Lyapunov exponent, therefore at or near a transition to or out of chaos. We give explicit examples ranging from exponential to logarithmic behavior, including Zipf’s law. Adoption of the nonlinear map as the formalism central character is a useful viewpoint, as variation of its few parameters, that modify its tangency property, translate into the different classes for *N*(*k*).

## Introduction

**Fig 1:**
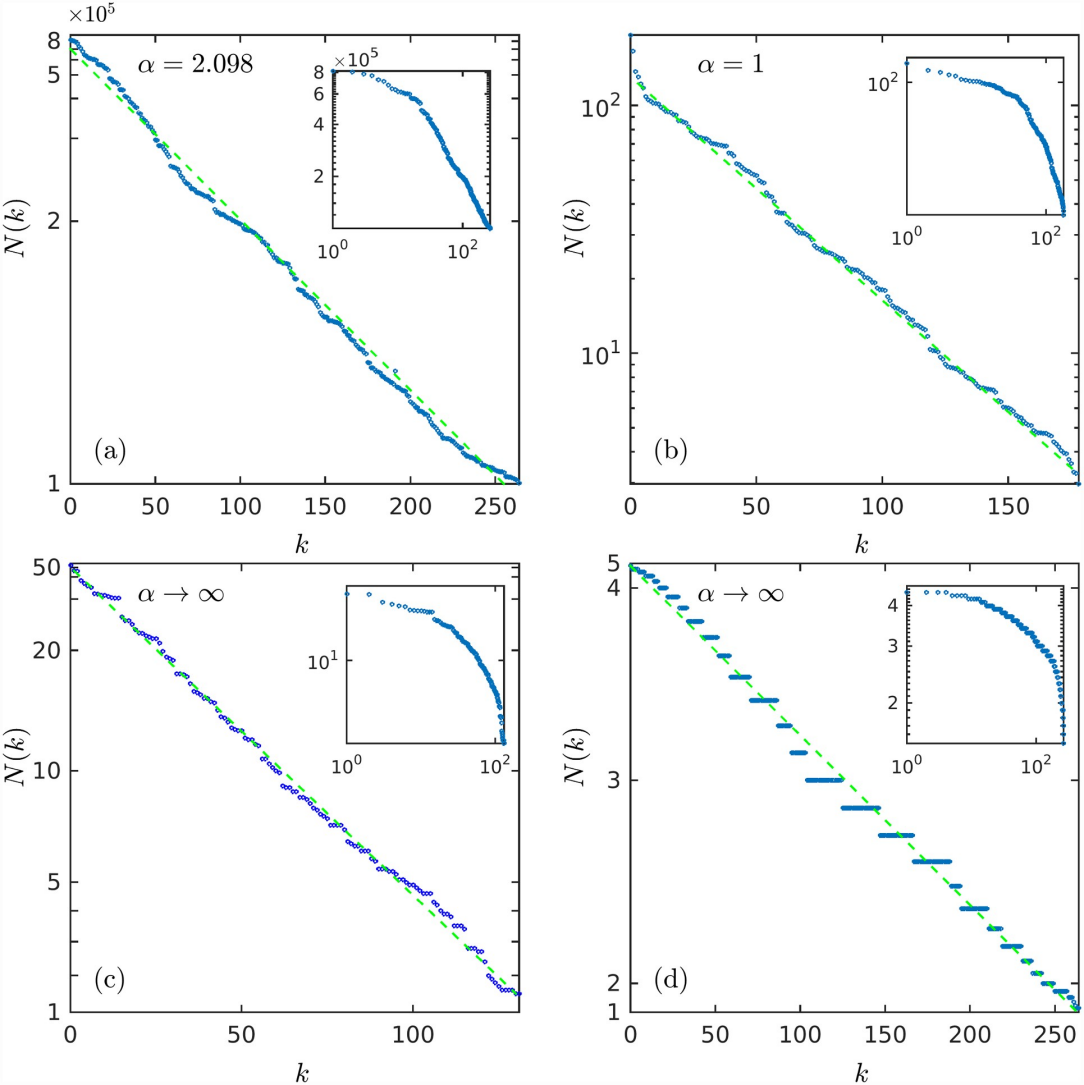
Ranked size data. Data *N*(*k*) plotted in scales such that they appear fitted by decreasing straight lines. (a) USA city populations [10], ordinates in ln_*α*_(*N*(*k*)/*N*_max_) scale. (b) Infant mortality per country [11], ordinates in logarithmic scale. (c) Firearms owned per 100 capita and per country [12], ordinates in −exp(− *N*(*k*)/*N*_0_) scale (*N*_0_ = 11.77). (d) Los Angeles household sizes [13], ordinates in $\text{erf}(\left(N\left(k\right)-\mu \right)/\left(\sqrt{2}\sigma \right))$ scale (*μ* = 2.97, *σ* = 0.644). The insets show the same data in logarithmic scales. See text for explanations, definitions and notation.

There exist countless sets of data detailing magnitudes or sizes of a vast variety of measurable properties from many different fields: astrophysical, geophysical, ecological, biological, technological, financial, urban, social, etc. The magnitude data sets can be ranked and after examination the resultant distributions can be classified into different families or groups according to the functional expression that fits them best, power law, exponential, logarithmic, inverse error function, etc. Fig 1 exemplifies four such types of distributions. Over the years, [1–7] interest has been mostly placed on ranked data that exhibits power-law behavior even if this is not observed over the entire collection of records since this suggests a possible relationship with the famous empirical Zipf’s law [8, 9]. Less attention has been placed to the comprehensive study of a wider range of types of rank distributions with the purpose of uncovering the broad-spectrum physics, if there is one, behind rank distributions. Here we consider this enterprise.

**Fig 2:**
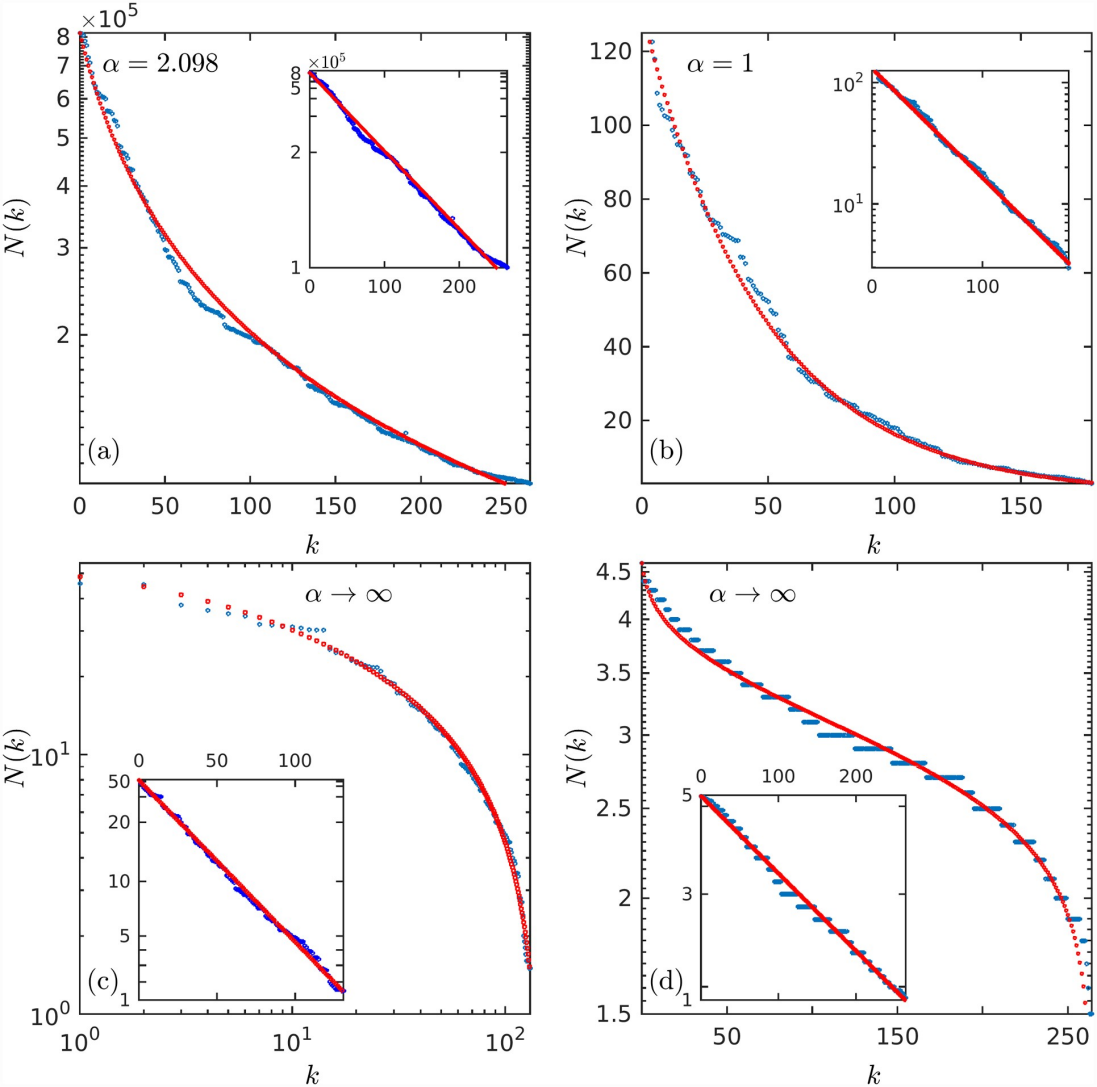
Data fitting. Another look at the fitting of data in Fig 1. (a) USA city populations, ordinates in ordinary logarithmic scale. (b) Infant mortality per country in ordinary linear scales. (c) Fireams owned per 100 capita and per country, in ordinary logarithmic scales, (d) Los Angeles household sizes, ordinates in ordinary logarithmic scale. The straight lines in the insets are obtained by using the same scales as those in Fig 1 or in the insets of Fig 3. See text for explanations, definitions and notation.

We start by recalling [3] a simple stochastic approach to reproduce size-rank data from an assumed parent distribution that governs the values of random variables that form finite samples. For every assumed form of the parent distribution one obtains a size-rank distribution. And this leads, when matched successfully with real data, to different possible families or universality classes. As an illustration we show (see Fig 1) four examples of data fitted by power law, exponential, logarithmic, and inverse error function expressions. The examples in Fig 1, and also further down in the text in Fig 2, correspond, respectively, to data on USA city populations [10], infant mortality [11], gun ownerships [12], and Los Angeles occupants of households [13]. The ordinates in Fig 1 and the ordinates in the insets in Fig 2 use scales such that the data approximate straight decreasing lines.

**Fig 3:**
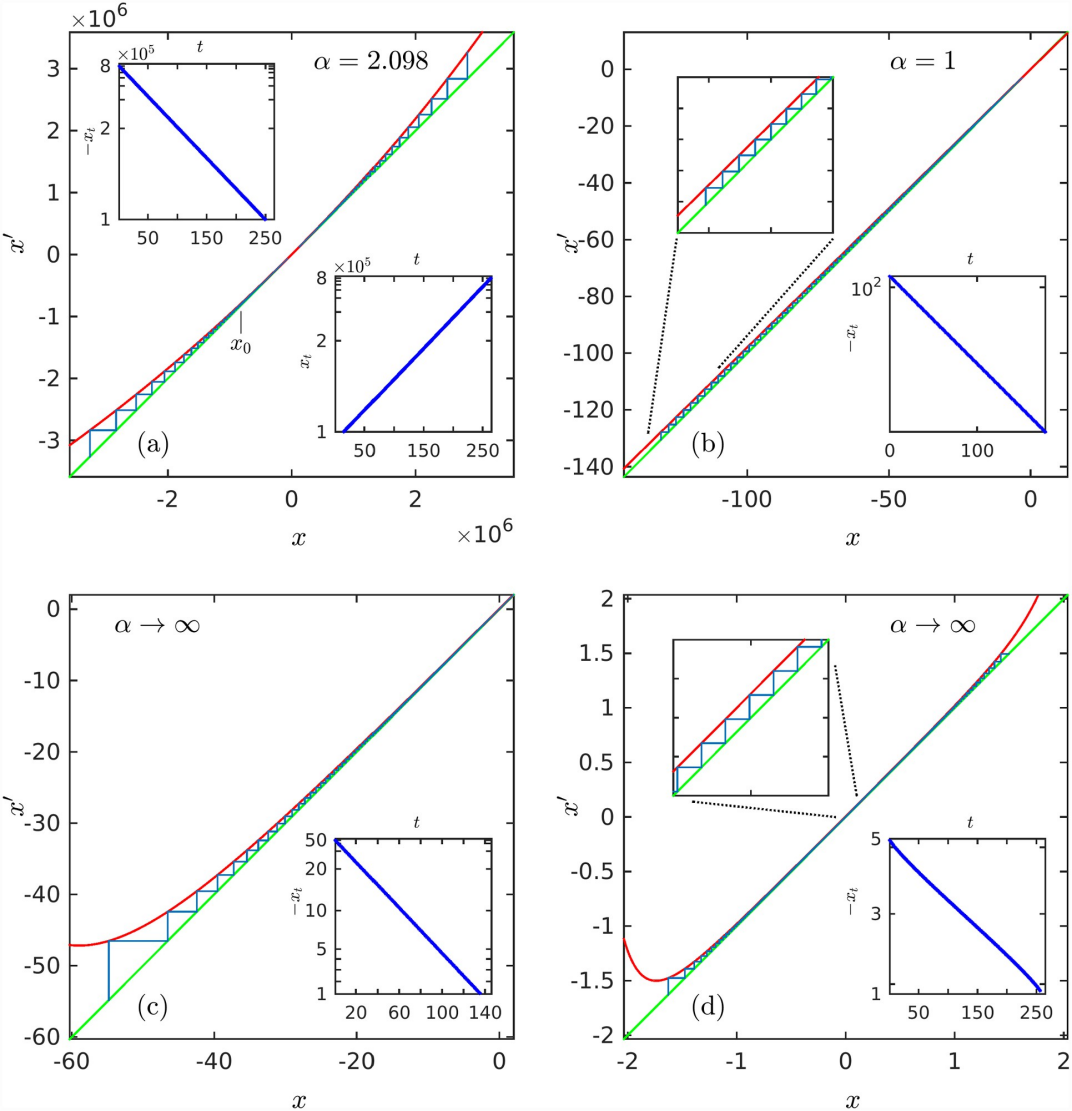
Nonlinear iterated maps and trajectories. Maps and trajectories that fit the data shown in Fig 1. The insets show the trajectories as (iterated) time series. (a) *x*′ = *x* exp_*z*_(*ux*^*z*−1^), inset ordinates in ∓ ln_*z*_ |*x*_*t*_/*x*_0_| scale. (b) *x*′ = (1 − *a*)*x*, inset ordinates in logarithmic scale. (c) *x*′ = *x* + *u* exp(− *x*/*N*_0_)*N*_0_, inset ordinates in −exp(− *x*/*N*_0_) scale. (d) $x^{\prime }=x+u\sqrt{2\pi }\sigma \exp{\left(\left(x-\mu \right)/\left(\sqrt{2}\sigma \right)\right)}^{2}$, inset ordinates in $\text{erf}(\left(-x_{t}-\mu \right)/\left(\sqrt{2}\sigma \right))$ scale. See text for explanations, definitions and notation.

After this, we translate the stochastic approach into a deterministic one by deriving nonlinear one-dimensional iterated maps such that their trajectories match exactly the aforementioned classes of size-rank distributions. In Fig 3 we plot the maps that correspond to the same four examples in Figs 1 and 2 and where the insets show the trajectories plotted with the same scales as before such that they become straight decreasing lines. We find that these maps have as a common feature being close to or at tangency with the identity line and therefore their trajectories have zero or small Lyapunov exponent as in the transition in or out of chaos via the tangent bifurcation [14].

Consideration of the nonlinear map as the central object in the formalism offers a unifying viewpoint. As we shall see, variation of its nonlinearity takes the map from intersection (with the identity line) to tangency, then to a shift of the tangency position to infinity, or to a shape with a central sector parallel and close to tangency. These circumstances translate, respectively, into different expressions for the rank distribution, first the mentioned exponential, then power law, next logarithmic, or inverse error function forms. We also present a novel use of the perturbed Renormalization Group (RG) fixed-point map of Hu and Rudnick [15] for the description of finite-size samples.

Finally, we indicate that the reciprocal of each value of the size-rank distribution (ranked magnitudes) can be used to define uniformly-distributed probabilities, one for each fixed value of the rank. And these in turn can be used to determine entropies. We discuss the occurrence of generalized entropy or information measure expressions in the limit when the Lyapunov exponent vanishes [16, 17]. These measures possess the extensivity property, where system (trajectory or sample) size is quantified by either final iteration time or maximum rank.

Originally [9], Zipf law referred to the number of occurrences of words in texts and since then it has been assigned also to the number of occurrences of other items and more freely to magnitudes or sizes of other entities. A large number of studies on the subject have been developed since and general considerations made, a taster is given in Refs [1–7]. Here we refer only to ranked magnitude or size data. For clarity of presentation a discussion on the distinction between the two kinds of rank distributions, frequency and magnitude, is not included here but can be found in [18].

## A stochastic approach to size-rank distributions

The starting quantity in our formalism is the probability distribution *P*(*N*) of the data *N* under consideration. This could have any form, we start by making use of the power law form [3, 18–20],

$$\begin{array}{c} P\left(N\right)∼N^{-\alpha },\;1\leq \alpha <\infty . \end{array}$$ (1)

A set of data, or an ensemble of them, can be extracted from Eq (1) to be compared with real data sets. We shall be interested in size-rank distributions so that the values of *N* represent the magnitude or size of the items in the data set, and therefore *N* takes positive values within the interval *N*_min_ ≤ *N* ≤ *N*_max_, with possibly *N*_min_ = 0 and/or *N*_max_ → ∞. We use N for the total number of data obtained from *P*(*N*).

The N items in the data set can be ordered or sorted out starting with the largest, *N*_max_, and proceeding with decreasing sizes down to *N*_min_. We label them with a new variable *k*, the rank, with *k* = 0 for *N*_max_ and *k* = *k*_max_ for *N*_min_. We call the function *N*(*k*) the size-rank distribution. The rank *k* can be an integer *k* = 0, 1, 2, 3, …, *k*_max_ (frequently the 1st value is *k* = 1) but it can be extended to be a real number. The main task is to determine *N*(*k*) from *P*(*N*).

Following [3] a well-known procedure to sort out random variable data [21], the next step is to introduce the (complementary) cumulative distribution of *P*(*N*) as

$$\begin{array}{c} \Pi \left(N,N_{\max}\right)=\int_{N}^{N_{\max}}P\left(N\right)dN, \end{array}$$ (2)

so that normalization of *P*(*N*) means Π(*N*_min_, *N*_max_) = 1. It is through the distribution Π(*N*, *N*_max_) that the set of data generated by *P*(*N*) is ordered by size to deliver *N*(*k*). As *N* is varied from *N*_max_ all the way down to *N*_min_ the distribution Π(*N*, *N*_max_) increases monotonically taking values from Π(*N*_max_, *N*_max_) = 0 to Π(*N*_min_, *N*_max_) = 1, and this distribution can be identified with *k* if properly scaled, that is

$$\begin{array}{c} k\equiv N\Pi (N\left(k\right),N_{\max}). \end{array}$$ (3)

The dependence of *N* on *k* can be obtained by solving

$$\begin{array}{c} \frac{k}{N}=\int_{N(k)}^{N_{\max}}P\left(N\right)dN, \end{array}$$ (4)

for *N*(*k*). We call *N*(*k*) the size-rank distribution even though it is not a probability distribution [18], as *P*(*N*) and Π(*N*, *N*_max_) are. If *P*(*N*) requires normalization, it must be divided by the constant

$$\begin{array}{ccc} C & = & \int_{N_{\min}}^{N_{\max}}P\left(N\right)dN, \end{array}$$ (5)

and Eq (4) generalises to

$$\frac{k}{N}=\frac{1}{C}\int_{N(k)}^{N_{\max}}P\left(N\right)dN.$$

Defining $N^{\prime }\equiv N/C$ we have

$$\begin{array}{c} \frac{k}{N^{\prime }}=\int_{N(k)}^{N_{\max}}P\left(N\right)dN. \end{array}$$ (6)

## Different classes of size-rank distributions

We look now at the specific expressions that come out of Eq (4) when *P*(*N*) is given by Eq (1). We have

$$\begin{array}{ccc} \Pi (N\left(k\right),N_{\max}) & = & \frac{1}{C_{\alpha }}\int_{N(k)}^{N_{\max}}N^{-\alpha }dN\\ & = & \frac{1}{C_{\alpha }\left(1-\alpha \right)}[N_{\max}^{1-\alpha }-N{\left(k\right)}^{1-\alpha }]\\ & = & \frac{1}{C_{\alpha }}\left[\ln_{\alpha }N_{\max}-\ln_{\alpha }N\left(k\right)\right], \end{array}$$ (7)

where ln_*q*_(*x*) ≡ (1 − *q*)^−1^[*x*^1−*q*^ − 1] with *q* a positive real number is the *q*-deformed logarithmic function, and *C*_*α*_ = ln_*α*_ *N*_max_ − ln_*α*_ *N*_min_. From Eqs (6) and (7) it follows

$$\begin{array}{c} \ln_{\alpha }N\left(k\right)=\ln_{\alpha }N_{\max}-N^{\prime -1}k. \end{array}$$ (8)

The normalization constant *C*_*α*_ = is finite if *N*_min_ > 0 and *N*_max_ < ∞. When *α* > 1 *C*_*α*_ is finite when *N*_max_ → ∞ but *N*_min_ > 0.

The size-rank distribution *N*(*k*) is explicitly obtained from the above with use of the inverse of ln_*q*_(*x*), the *q*-deformed exponential function exp_*q*_(*x*) ≡ [1 + (1 − *q*)*x*]^1/(1 − *q*)^, this is

$$\begin{array}{c} N\left(k\right)=N_{\max}\exp_{\alpha }\left[-N_{\max}^{\alpha -1}N^{\prime -1}k\right], \end{array}$$ (9)

so that when *α* = 1 we have the ordinary exponential form

$$\begin{array}{c} N\left(k\right)=N_{\max}\exp\left(-N^{\prime -1}k\right). \end{array}$$ (10)

We can take the limit *α* → ∞ to signify, for instance, that

$$\begin{array}{c} P\left(N\right)=N_{0}^{-1}\exp\left(-N/N_{0}\right), \end{array}$$ (11)

and we choose *N*_0_ = 1 to find

$$\begin{array}{c} N\left(k\right)=-\ln[\exp\left(-N_{\max}\right)+N^{\prime -1}k]. \end{array}$$ (12)

In the limit *N*_max_ → ∞ Eq (9) becomes the power law *N*(*k*) ∼ *k*^1/(1−*α*)^ that when *α* = 2 gives the simple hyperbolic form *N*(*k*) ∼ *k*^−1^, the classical Zipf law for ranked size data. In contrast, when *α* → ∞, in the limit *N*_max_ → ∞ the rank distribution becomes $N\left(k\right)=-\ln\left(k/N^{\prime }\right)$, *N*(*k*) decays very fast as *k* increases since the argument in the logarithmic function lies in the interval $0\leq k/N^{\prime }\leq 1$. This can be compared with the case *α* = 1, but *N*_max_ finite, when *N*(*k*) decays exponentially as *k* increases.

Another, important, option for *P*(*N*) for the limit when *α* → ∞ is the Gaussian distribution

$$\begin{array}{c} P\left(N\right)=\frac{1}{\sqrt{2\pi }\sigma }\exp(-\frac{{\left(N-\mu \right)}^{2}}{2\sigma^{2}}), \end{array}$$ (13)

for which we find

$$\begin{array}{c} N\left(k\right)=\mu +\sqrt{2}\sigma \;{\text{erfc}}^{-1}[\text{erfc}(\frac{N_{\text{max}}-\mu }{\sqrt{2}\sigma })+2kN^{\prime -1}]. \end{array}$$ (14)

The data in the panels of Fig 1 are plotted using scales such that *N*(*k*) given by Eqs (9), (10), (12) and (14) appear as decreasing straight lines.

## Nonlinear dynamical analogues of size-rank distributions

The stochastic procedure described above to obtain size-rank functions can be transformed into a deterministic one where trajectories *x*_*t*_, *t* = 0, 1, … of a one-dimensional nonlinear iterated map *x*′ = *f*(*x*), reproduce the functions *N*(*k*), *k* = 0, 1, …. The general expression for this map is

$$\begin{array}{c} f(x)=x+u/P(-x),u>0, \end{array}$$ (15)

where *P*(− *x*) ≥ 0 is, as above, the parent distribution and $u=N^{\prime -1}$. To obtain Eq (15) we differentiate Eq (6) to give

$$\begin{array}{c} -\frac{1}{N^{\prime }}\frac{dk(N)}{dN}=P\left(N\right), \end{array}$$ (16)

or, in terms of dynamical variables,

$$\begin{array}{c} u\frac{dt}{dx_{t}}=P\left(-x_{t}\right). \end{array}$$ (17)

Use of *dx*_*t*_/*dt* ≃ *x*_*t*+1_ − *x*_*t*_ recovers Eq (15).

The size-rank distributions *N*(*k*) obtained from the trajectories *x*_*t*_ of any map *f*(*x*) in our procedure are monotonically decreasing functions of its rank *k*. This is so if all trajectories *x*_*t*_ generated by *f*(*x*) are monotonically increasing functions of the iterated time *t*, as *N* = −*x* and *k* = *t*. This is so because the condition *f*(*x*) ≥ *x* is always satisfied by Eq (15). Notice that *f*(*x*) (or *P*(*N*)) may not be monotonic functions themselves.

This exact analogy between the expressions for the rank distributions and those for the dynamics in a one-dimensional map was recognized before for the special case of the power law parent distribution in Eq (1) [18–20], where the map is at a tangent bifurcation [14]. In this case *f*(*x*) is written locally as

$$\begin{array}{c} x^{\prime }=f\left(x\right)=x+u{\left|x\right|}^{z}+\cdots ,\;x\leq 0,\;z>1, \end{array}$$ (18)

and trajectories initiated at *x*_0_ ≲ 0 are obtained from

$$\begin{array}{c} x_{\tau +1}=x_{\tau }+u{\left|x_{\tau }\right|}^{z},\;\tau =0,1,\ldots \end{array}$$ (19)

These trajectories move monotonically towards the point of tangency at *x* = 0. If we make the replacement, valid for large time *τ*, of the difference *x*_*τ*+1_ − *x*_*τ*_ by *dx*_*τ*_/*dτ* in Eq (19) (written as *u*|*x*_*τ*_|^*z*^ = *x*_*τ*+1_ − *x*_*τ*_) we obtain the differential form *udτ* = |*x*_*τ*_|^−*z*^ *dx*_*τ*_, and integration of both sides of it yields

$$\begin{array}{ccc} ut & = & \int_{x_{0}}^{x_{t}}\frac{dx_{\tau }}{|x_{\tau }{|}^{z}}=\int_{-x_{t}}^{-x_{0}}\frac{dx_{\tau }}{|x_{\tau }{|}^{z}}\\ & = & \frac{1}{1-z}[|x_{0}{|}^{1-z}-{\left|x_{t}\right|}^{1-z}], \end{array}$$ (20)

or

$$\begin{array}{c} \ln_{z}|x_{t}|=\ln_{z}\left|x_{0}\right|-ut. \end{array}$$ (21)

The iteration number or time *t* dependence of all trajectories is obtained by solving the above for *x*_*t*_, i.e.

$$\begin{array}{c} x_{t}=x_{0}\exp_{z}[x_{0}^{z-1}ut], \end{array}$$ (22)

where *x*^*z*−1^ ≡ sign(*x*) |*x*|^*z*−1^. The equivalence of the trajectory positions *x*_*t*_ with the size-rank distribution *N*(*k*) is made clear by comparison of Eqs (21) and (22) with Eqs (8) and (9), respectively, together with the identifications *t* = *k*, $u=N^{\prime -1}$, *x*_*t*_ = −*N*(*k*), *x*_0_ = −*N*_max_ and *z* = *α*. When *z* = 1 Eq (22) becomes an ordinary exponential,

$$\begin{array}{c} x_{t}=x_{0}\exp\left(-at\right), \end{array}$$ (23)

that matches, with use of the previous identifications, Eq (10). As we see below, a linear map that intersects the identity line,

$$\begin{array}{c} x^{\prime }=f\left(x\right)=\left(1-a\right)x, \end{array}$$ (24)

reproduces the trajectories in Eq (23).

In establishing the equivalence between trajectories *x*_*t*_ and rank functions *N*(*k*) when 1 < *α* = *z* < ∞ we used starting positions at the left of the point of tangency *x*_0_ < 0, but we could have obtained the same monotonically decreasing *N*(*k*) from starting positions at the right of the tangency point *x*_0_ > 0 with the use of the identifications *k* = *T* − *t*, *x*_*T*_ = *N*_max_, *x*_*t*_ = *N*(*k*), where *T* is the last iteration time.

We call attention to the fact [22] that the map trajectories given by Eq (22) are precisely those that are generated by the renormalization group (RG) fixed-point map [14, 15]

$$\begin{array}{c} x^{\prime }=f\left(x\right)=x\exp_{z}\left(ux^{z-1}\right), \end{array}$$ (25)

where this map was obtained [14, 15] as the solution of the functional composition RG fixed point equation

$$\begin{array}{c} f\left(f\left(x\right)\right)=\gamma^{-1}f\left(\gamma x\right), \end{array}$$ (26)

with *γ* = 2^*z*−1^ and where the expansion of *f*(*x*) starts as the required local form for tangency *f*(*x*) = *x* + *u*|*x*|^*z*^ + ⋯, *x* ≤ 0, *z* > 1.

In the limit *z* → ∞ the counterpart of Eq (22) is

$$\begin{array}{c} x_{t}=\ln[\exp(x_{0})+ut], \end{array}$$ (27)

as this expression transforms into Eq (12) for *N*(*k*) under the same equivalences *t* = *k*, $u=N^{\prime -1}$, *x*_*t*_ = −*N*(*k*), *x*_0_ = −*N*_max_. Differentiation of Eq (27) gives

$$\begin{array}{c} \frac{dx_{t}}{dt}=u\exp\left(-x_{t}\right), \end{array}$$ (28)

so that use of *x*_*t*+1_ − *x*_*t*_ ≃ *dx*_*t*_/*dt*, *t* ≫ 1, yields the map

$$\begin{array}{c} x^{\prime }=x+u\exp\left(-x\right). \end{array}$$ (29)

We note that the tangency of this map with the identity line is located at *x* → ∞ and not at *x* = 0 as it is the case of the map in Eq (25), the effect of taking *z* = *α* → ∞ has shifted the tangency position. To obtain the trajectories *x*_*t*_ in Eq (27) from the map in Eq (29) it is necessary to perform a coordinate transformation to bring the point of tangency back to *x* = 0.

We can follow the same procedure for the above cases to determine the map that corresponds to the Gaussian distribution choice for *P*(*N*) and, consequently, the inverse complementary error function for *N*(*k*). This is

$$\begin{array}{c} x^{\prime }=x+u\sqrt{2\pi }\sigma \exp(\frac{{\left(x-\mu \right)}^{2}}{2\sigma^{2}}). \end{array}$$ (30)

This map never touches the identity line but it comes increasingly closer and parallel to this line as *u* → 0. The trajectories generated by the map in Eq (30) are given by

$$\begin{array}{c} x_{t}=-\mu -\sqrt{2}\sigma \;{\text{erfc}}^{-1}[\text{erfc}(\frac{-x_{0}-\mu }{\sqrt{2}\sigma })+2ut]. \end{array}$$ (31)

In Fig 3 we show the maps and their trajectories that correspond to the four cases described here, those in Eqs (25), (24), (29) and (30). The insets in the panels of Fig 3 are plotted using scales such that the trajectories *x*_*t*_ given by Eqs (22), (23), (27) and (31) appear as decreasing straight lines. An exception is the bottom inset of panel (a) that corresponds to an initial position placed at the right of the point of tangency. In Fig 2 we reproduce the data in the panels of Fig 1 using the same scales as before so that the straight lines correspond to the maps trajectory positions −*x*_*t*_ = *N*(*k*), *t* = *k*.

## A closer look at rank distributions for finite-size samples

As mentioned, for finite data sets the distribution *P*(*N*) cannot be a pure power law as in Eq (1). We use the analogy with the map at tangency to deal with this circumstance without the need to find the explicit form for *P*(*N*). When the tangent RG fixed-point map in Eq (25) is perturbed by a small amount *ε* one obtains [15]

$$\begin{array}{ccc} \;f(x,\varepsilon ) & = & x\exp_{z}[ux^{z-1}]\\ & & \;+\;\frac{\varepsilon }{z-1}x^{-(z-1)}(1-\left(z-1\right)ux^{z-1}-\exp_{z}{\left[ux^{z-1}\right]}^{z})+O\left(\varepsilon^{2}\right). \end{array}$$ (32)

**Fig 4:**
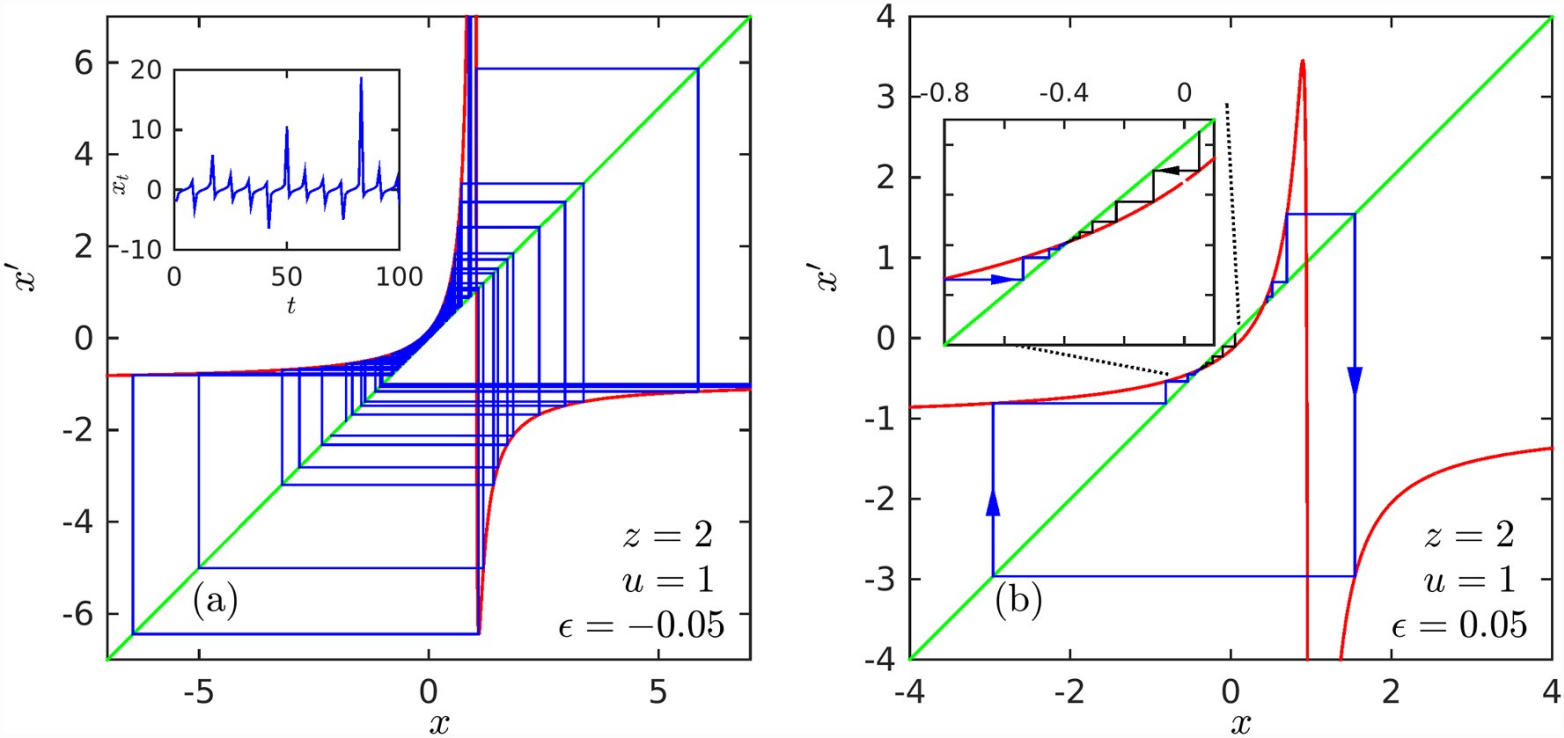
Perturbed RG fixed-point maps. (a) *ϵ* < 0. A chaotic trajectory is shown performing passages through the map narrow channel close to the identity line mediated by reinjections that make use of the bottom branch of the map that occurs after the map singularity at 0. (b) *ϵ* > 0. The intersections of the map with the identity line, attractor and repellor positions, generate trajectories that are guided towards the attractor and/or driven away from the repellor.

According to the sign of *ε* the map in Eq (25) is shifted off tangency letting trajectories progress across a narrow channel (see Fig 4(a)), or producing a bisection of the map with the identity line (see Fig 4(b)). The effect on the rank distribution *N*(*k*) can be observed by use of the identifications given above between nonlinear map and rank distribution variables to find the appropriate results for N<∞. In doing this the dependence of *x*_*t*_ on the shift parameter *ε* is translated into *N*(*k*), and one immediate way of assessing its relevance to the description of finite N<∞ samples is through direct comparison.

When *ε* > 0 the map intersects the identity line *x*′ = *x* twice, say at *x*_*l*_ < 0 and *x*_*r*_ > 0. The map in the neighborhood of *x*_*l*_ has the form *f*(*x*) = *x* − *ax* + ⋯, where the variable *x* has been redefined by a shift such that *x* = 0 is the old *x*_*l*_, and trajectories close to *x*_*l*_ evolve as *x*_*t*+1_ = *x*_*t*_ − *ax*_*t*_ + ⋯. The differential version of the latter is $a_{}dt=-x_{t}^{-1}dx_{t}$, so that, integration of both sides of it yields −ln(*x*_*t*_/*x*_0_) = *at*, or

$$\begin{array}{c} x_{t}=x_{0}\exp\left(-at\right),\;x_{0}<0. \end{array}$$ (33)

This is the map equivalent to the size-rank distribution *N*(*k*) given in Eq (10) for the case when *α* = 1 (where again *t* = *k*, $a=N^{\prime -1}$, *x*_*t*_ = −*N*(*k*) and *x*_0_ = −*N*_max_). Therefore the effect of a positive shift *ε* > 0 is to transform the power law *P*(*N*) ∼ *N*^−*α*^, *α* > 1, by the hyperbolic form *P*(*N*) ∼ *N*^−1^ as *N* increases. When *ε* > 0 is very small the map is very close to tangency, the intersection *x*_*l*_ ∼ 0, and a crossover should be observed in *N*(*k*) from power law *k*^−*α*^ to exponential decay.

In establishing the equivalence between the trajectory *x*_*t*_ and the rank function *N*(*k*) we used starting positions at the left of the intersection *x*_*l*_ < 0, but we could have obtained the same monotonically decreasing *N*(*k*) from starting positions at the right of the intersection *x*_*r*_ > 0, after a shift such that *x* = 0 is the old *x*_*r*_ and with the use of the identifications *k* = *T* − *t*, *x*_*T*_ = *N*_max_, *x*_*t*_ = *N*(*k*), where *T* is the last iteration time.

**Fig 5:**
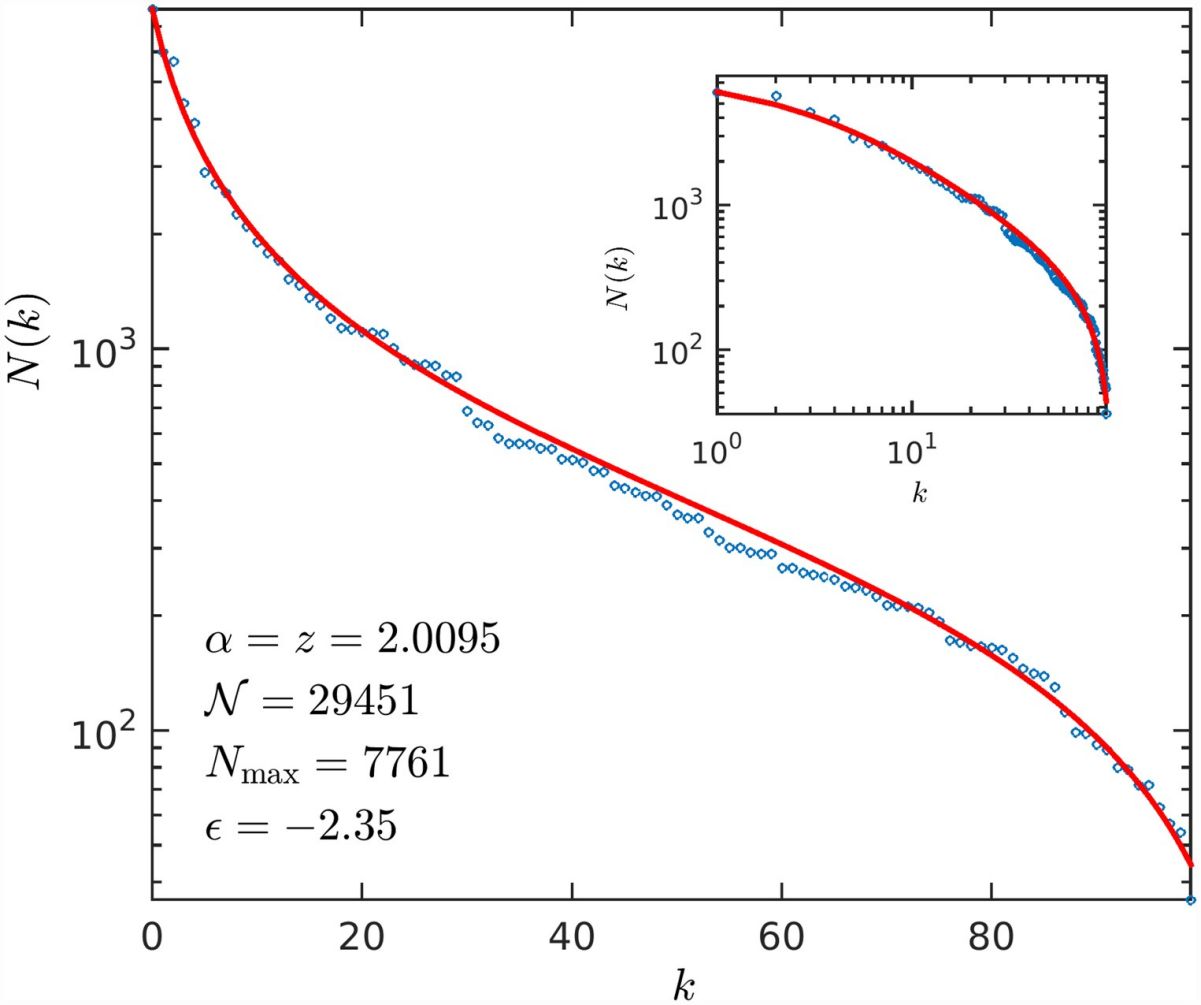
Rank-order statistics for cities with largest numbers of tallest buildings. Data (empty circles) from Reference [23]. Fitting obtained from a trajectory (smooth curve) of the map in Eq (32) with the identifications provided in the text.

When *ε* < 0 the map does not intersect the identity line *x*′ = *x* but there is a narrow channel through which trajectories started at *x*_0_ < 0 slip into positions *x*_*t*_ > 0. Once a trajectory traverses the channel it increases its value *x*_*t*_ rapidly, and this behavior is reflected in *N*(*k*) as a sharp bend towards *N* = 0 reaching this value at a finite rank, *k*_max_ < ∞. In this way the outcome of a negative shift *ε* < 0 is a finite size effect. The transformation of *x*_*t*_ into *N*(*k*) follows the earlier identifications *t* = *k*, $u=N^{\prime -1}$, and *z* = *α*, but now *x*_0_ − x* = −*N*_max_ and *x*_*t*_ − x* = −*N*(*k*), where the translation x* ensures that all *N*(*k*) ≥ 0. In particular, for a map with parameters (*z*, *u*) and given value of *ε*, a trajectory started at *x*_0_ and recorded until a certain maximum value *x*_*T*_ at time *T* before escaping from the narrow channel we have x* = *x*_*T*_ and *T* = *k*_max_. We show in Fig 5 (see inset) that this indeed is observed in real data. We used data in this particular case of numbers of tallest buildings [23].

Notice that for the map at tangency, *ε* = 0, any trajectory with *x*_0_ < 0 evolves towards *x* = 0 asymptotically as a power law *t*^1/(1−*z*)^, *z* > 1, reaching *x* = 0 at *t* → ∞. This implies $k_{\max}=N\to \infty$, and signifies infinite size or a ‘thermodynamic limit’ as suggested in the following Section. The shift off tangency *ε* < 0 translates into $k_{\max}=N<\infty$ for the rank distribution.

Chaotic dynamics arises via the intermittency property of trajectories when *ϵ* = 0 turns *ϵ* ≲ 0 (inset of Fig 4a). The passages through the narrow channel are the so-called laminar episodes (monotonic curves) separated by re-injections (that break monotonicity). One such laminar episode was used to fit the data in Fig 5. The duration *D* of the laminar episodes depends on the value of *ϵ* (*D* diverges as *ϵ* vanishes as a known power law [14]). In the analogy *D* equals the maximum value of the rank *k*_max_, the size of the data sample. This connection provides a means to treat finite size without the need to determine the appropriate parent distribution *P*(*N*) that is required to replace the pure power law (strictly compatible only with infinite size) *P*(*N*) in Eq (1). A sequence of laminar episodes (as shown in the inset of Fig 4a) provides an ensemble of finite-size data samples.

## A statistical-mechanical structure for size-rank distributions

As pointed out before [18–20] the size-rank expressions such as those in Eqs (8) and (9) are reminiscent of those in a statistical-mechanical and thermodynamic structure. First consider that for a fixed value of *k* the probability for each unit of *N* that makes up *N*(*k*) to occur is $p_{i}^{\left(k\right)},i=1,\ldots ,N\left(k\right)$. If this probability is uniform as in a microcanonical ensemble, we have $p_{i}^{\left(k\right)}=p^{\left(k\right)}\equiv 1/N\left(k\right)$ for all *i* = 1, …, *N*(*k*), and this for each *k* = 0, 1, …, *k*_max_. Then, if we consider the case *α* = 1 and define *S*(*k*) ≡ ln *N*(*k*) and *S*_max_ ≡ ln *N*_max_, Eq (8) reads

$$\begin{array}{c} S\left(k\right)=S_{\max}-N^{\prime -1}k, \end{array}$$ (34)

where if $S_{\max}\left(N^{\prime -1}\right)$ is thought of as an entropy for the system with fixed $N^{\prime }$ then *S*(*k*) would be a Massieu potential [24] after the variable $N^{\prime -1}$ has been replaced by the (conjugate) variable *k* as in a Legendre transformation. Eq (10),

$$\begin{array}{c} N\left(k\right)=N_{\max}\exp\left(-N^{\prime -1}k\right), \end{array}$$ (35)

can be taken to be the outcome of the steepest-descent property arising for large $N^{\prime }$ on

$$\begin{array}{c} N\left(k\right)=\int N_{\max}\left(N^{\prime }\right)\exp\left(-N^{\prime -1}k\right)dN^{\prime -1}. \end{array}$$ (36)

That is, we consider that there are fluctuations in the data sample size $N^{\prime }$, and consequently also in the degeneration $N_{\max}\left(N^{\prime }\right)$, but these become increasingly irrelevant for large $N^{\prime }$ as the limit $k_{\max}=N\to \infty$, and *N*_min_ → 0, and a dominant configuration is established in analogy with the thermodynamic limit.

The case *α* > 1, where now *S*(*k*) ≡ ln_*α*_ *N*(*k*) and *S*_max_ ≡ ln_*α*_ *N*_max_, together with the use of exp_*α*_ instead of the ordinary exp in Eqs (35) and (36) demands careful analysis of the limit $k_{\max}=N\to \infty$. It has been argued recently [16, 17] that this type of generalized expression for the entropy appears in situations where a drastic reduction of accessibility to configuration space is forced upon a system, such that the final set of reachable configurations has a vanishing measure with respect to the initial set. Attractors in dissipative nonlinear systems provide a mechanism for such severe reduction of accessible states, and the case of the tangent bifurcation, as other transitions to chaos in low-dimensional iterated maps, have proved to be suitable model systems to analyze this circumstance [16, 17]. Interestingly, the RG fixed-point map Eq (25) provides a simple analytical expression that illustrates both this phase space (or position interval) contraction (from a set of real numbers to only a finite set) and a connection with the size-rank distribution *N*(*k*) in Eq (9).

The above entropy expressions form part of a set of extensive entropy (or information) measures that comprise the four types of distributions *N*(*k*) we have described (Eqs (9), (10), (12) and (14) and shown in Fig 2). These measures are given by their corresponding unnormalized cumulative distributions $N\Pi (N\left(k\right),N_{\max})$:

$$\begin{array}{c} N\Pi \left(N\left(k\right),N_{\max}\right)=k_{\max}[\ln N_{\max}-\ln N\left(k\right)],\;\alpha =1, \end{array}$$ (37)

$$\begin{array}{c} N\Pi \left(N\left(k\right),N_{\max}\right)=k_{\max}[\ln_{\alpha }N_{\max}-\ln_{\alpha }N\left(k\right)],\;1<\alpha <\infty , \end{array}$$ (38)

$$\begin{array}{c} N\Pi \left(N\left(k\right),N_{\max}\right)=k_{\max}[\exp\left(N\left(k\right)\right)-\exp\left(N_{\max}\right)],\;\alpha \to \infty , \end{array}$$ (39)

and

$$\begin{array}{cc} 2N\Pi (N\left(k\right),N_{\max}) & =k_{\max}[\text{erfc}(\frac{N(k)-\mu }{\sqrt{2}\sigma })-\text{erfc}(\frac{N_{\text{max}}-\mu }{\sqrt{2}\sigma })],\;\alpha \to \infty . \end{array}$$ (40)

Since the size of the system is given by the rank *k*, 0 < *k* < *k*_max_ that generates the data set *N*(*k*), *k* = 0, 1, …, then by virtue of Eq (3) the above measures are extensive. In the nonlinear map language the measures are areas under the maps *f*(*x*) that grow linearly with iteration time (c.f. Eq (20)).

## Summary and discussion

We described different classes of size-rank distributions *N*(*k*) that originate each from a different parent distribution *P*(*N*) for data samples of the size random variable *N*. We considered power law, exponential, and Gaussian parent distributions to obtain analytic expressions for *N*(*k*). Then we derived for each of these cases expressions of one-dimensional nonlinear iterated maps such that their trajectories *x*_*t*_ are exact functional analogues of the size-rank distributions *N*(*k*), via *t* = *k*, *x*_*t*_ = −*N*(*k*), etc. Significantly, all the equivalent maps appear at or close to tangency with the identity line, as can be clearly seen in Fig 3, so that the Lyapunov exponent vanishes or is close to zero, and dynamical evolution takes place at or is close to a transition in or out of chaos via the tangent bifurcation. The Lyapunov exponent λ vanishes for the map in Eq (25) and is proportional to *ϵ* ≃ 0 for the perturbed map in Eq (32) [14, 22]. Straightforward calculations show that λ vanishes for the maps in Eq (29) (for all *u*) and Eq (30) (for *u* ≃ 0). Seen via the dynamical analogues there is a unifying thread that links these classes of rank functions: When the nonlinearity is *z* = 1 the map intersects the identity line and we have an exponentially-decaying *N*(*k*), Eq (10). When 1 < *z* < ∞ the map is tangent to the line and *N*(*k*) decays as a power law, Eq (9). When *z* → ∞ we observed that the point of tangency shifts to infinity and *N*(*k*) acquires a logarithmic form, Eq (12). Also, for the same *z* → ∞ the map can show a shape with a central sector parallel and close to tangency in which case *N*(*k*) exhibits an inverse error function form, Eq (14). The latter class represents the common situation in which the size variable *N* is controlled by the Central Limit Theorem. The description of rank functions in the alternative language of nonlinear dynamics opens the possibility of gains with the use of a different narrative. All one-dimensional nonlinear maps are dissipative so that they possess attractors and repellors and their known dynamical properties [14], some of which we have used here, may find further application. For all classes of size-rank distributions described we made comparison with real data and found agreement.

When the parent distribution is a power law, *P*(*N*)∼*N*^−*α*^, *α* > 1, the resulting map is that which corresponds to the fixed point of the RG approach where the basic transformation is functional composition, i.e. a deformed exponential map, c.f. Eq (25), which describes scaling at the tangent bifurcation. There, when the largest data *N*_max_ → ∞, *N*(*k*) becomes a power law *N*(*k*)∼*k*^1/(1−*α*)^, the classical Zipf law for *α* = 2. This is the most common case since then the local map at tangency is an analytic function with nonzero curvature. We have also argued about the statistical-mechanical meaning of the expressions we use to reproduce ranked data.

Significantly, the RG fixed-point map also appears as a central element in the description of critical fluctuations [25, 26] and helps determine their temporary, intermittent, nature. Also, the divergence of the critical correlation length implies the vanishing of the Lyapunov exponent at the tangent bifurcation [25, 26].

## References

[1] To Honor G.K. Zipf Glottometrics 3,4,5 Ludenscheid: RAM-Verl., 2002 ISSN 1617-8351. Available from: http://www.ram-verlag.eu/journals-e-journals/glottometrics/.

[2] Newman M.E.J. Power laws, Pareto distributions and Zipf’s law. Contemporary Physics 2005; 46(5):323–351. 10.1080/00107510500052444.

[3] Pietronero L. Tosatti E. Tosatti V. Vespignani A. Explaining the uneven distribution of numbers in nature: the laws of Benford and Zipf. Physica A. 2001; 293(1-2):297–304. 10.1016/S0378-4371(00)00633-6.

[4] Cristelli M. Batty M. Pietronero L. There is more than a power law in Zipf. Sci Rep 2012; 2(812):1–7. 10.1038/srep0082. PMC3492871 23139862

[5] Finley B.J. Kilkki K. Exploring empirical rank-frequency distributions longitudinally through a simple stochastic process. PLoS ONE 2014; 9(4):1–14. 10.1371/journal.pone.0094920. PMC3995693 24755621

[6] Moreno-Sánchez I. Font-Clos F. Corral A. Large scale analysis of Zifp’s law in English texts. PLoS ONE 2016; 11(1):1–19. 10.1371/journal.pone.0147073. PMC4723055 26800025

[7] Lestrade S. Unzipping Zipf’s law. PLoS ONE 2018; 12(8):1–13. 10.1371/journal.pone.0181987. PMC5549924 28792963

[8] van der Galien S.J.G. Zipf’s law. Wikipedia. Available from: http://en.wikipedia.org/wiki/Zipfs_law (2003).

[9] Zipf G.K. Human Behavior and the Principle of Least Effort. Cambridge: Addison-Wesley, 1949.

[10] USA city populations. The Rank-Size Rule of City Populations (USAcities2009.csv). Available from: http://www.statisticalconsultants.co.nz/blog/the-rank-size-rule-of-city-populations.html.

[11] Infant Mortality. NationMaster Geographical Data. Available from: http://www.nationmaster.com/country-info/stats/Health/Infant-mortality-rate.

[12] Estimated number of guns per 100 capita by country. Wikipedia. Available from: http://en.wikipedia.org/wiki/Number_of_guns_per_capita_by_country.

[13] Household Size. Los Angeles Times. Available from: http://projects.latimes.com/mapping-la/neighborhoods/household-size/neighborhood/list/.

[14] Schuster H.G. Deterministic Chaos. An Introduction. VCH Publishers, Weinheim; 1988.

[15] Hu B. Rudnick J. Exact solutions to the Feigenbaum renormalization- group equations for intermittency. Phys Rev Lett, 1982; 48: 1645–1648. 10.1103/PhysRevLett.48.1645.

[16] Yalcin G.C. Robledo A. Gell-Mann M. Incidence of q statistics in rank distributions. Proc Natl Acad Sci USA. 2014; 111(39):14082–14087. 10.1073/pnas.1412093111. 25189773 PMC4191817

[17] Yalcin G.C. Velarde C. Robledo A. Entropies for severely contracted configuration space. Heliyon, 2015; 1:e00045 Available from: http://www.heliyon.com/article/e00045/ 10.1016/j.heliyon.2015.e00045. 27441229 PMC4945624

[18] Velarde C. Robledo A. Rank distributions: Frequency vs. Magnitude. PLoS ONE 2017; 12(10):1–13. 10.1371/journal.pone.0186015. PMC5628998 28982160

[19] Altamirano C. Robledo A. Possible thermodynamic structure underlying the laws of Zipf and Benford. Eur Phys J B. 2011; 81(3):345–351. 10.1140/epjb/e2011-10968-5.

[20] Robledo A. Laws of Zipf and Benford, intermittency, and critical fluctuations. Chinese Sci Bull. 2011; 56(34):3645–3648. 10.1007/s11434-011-4827-y.

[21] Empirical cumulative distribution function. Wikipedia. Available form: https://en.wikipedia.org/wiki/Empirical_distribution_function.

[22] Baldovin F. Robledo A. Sensitivity to initial conditions at bifurcations in one-dimensional nonlinear maps: Rigorous nonextensive solutions. Eur Phys Lett. 2002; 60:518–524. http://stacks.iop.org/0295-5075/60/i=4/a=518.

[23] Skyline Ranking. Emporis. Available from: http://www.emporis.com/statistics/skyline-ranking.

[24] Callen H.B. Thermodynamics and an introduction to thermostatistics. Wiley, Cambridge; 1993.

[25] Robledo A. Unorthodox properties of critical clusters. Mol Phys. 2005; 103:3025–3030. 10.1080/00268970500185989.

[26] Riquelme-Galván M. Robledo A. Dual characterization of critical fluctuations: Density functional theory & nonlinear dynamics close to a tangent bifurcation. Eur Phys J S T. 2017; 226:341–351. 10.1140/epjst/e2016-60268-0.

